# Myocardial salvage after myocardial infarction depends on early therapy

**Published:** 2010-12

In a thought-provoking session on triple anti-platelet therapy in acute coronarysyndromes, Prof Marco Valgimigli,Ferrara, Italy, argued that the effect ofclopidogrel is negligible if given toolate to ST-segment elevation myocardialinfarction (STEMI) patients. ‘Clopidogrelis not fully absorbed during STEMI.This impaired bio-availability of both theactive and inactive metabolite,^1^ combinedwith late administration of clopidogrel, results in minimal anti-platelet inhibition. In fact, there is limited clinical evidencesupporting the upstream use of clopidogrel’, he said.

Referring to the BRAVE 3 study ofabciximab which, when added to aspirinand clopidogrel in STEMI patients, showed a negligible effect in further reducing infarct size and mortality. Prof Valgimigli ascribed this also to latedelivery of the study drug. This negativeoutcome was interpreted by the BRAVE 3 study investigators that abciximab should not be given to STEMI patients in the catheterisation laboratory.

‘In my view this is not the correct interpretation’, said Prof Valgimigli. ‘The problem was that the study drug was given after four hours to more than 50% of patients, and time from symptom onset to PCI was more than five hours in over 50% of the patients. If you consider the relationship between time-to-treatment reperfusion and the extent of myocardial salvage,^2^ this is not linear. While early reperfusion within at least four hours results in a large reduction in mortality following significant myocardial salvage, later intervention results in a much reduced mortality reduction. Therefore the overall lack of benefit from abciximab in Brave 3 is due to treatment and reperfusion delay.’

Using Medical Research Institute (MRI) studies^3^ of STEMI patients stratified by delay of treatment, Prof Valgimigli pointed out that the area of potential myocardial salvage is largest if time to reperfusion is short and within one to two hours post infarct. Therefore GIIb/ IIIa agents are of very significant value if given early.

The evidence for early administration of anti-platelet agents has been well shown in the ON-TIME-2 trial^2^ of very early upstream bolus tirofiban treatment. At the time of arrival at the PCI centre, patients treated with tirofiban on top of aspirin and clopidogrel had significantly lower residual ST-segment deviation than those receiving only aspirin and clopidogrel. The one-year survival rate of those patients receiving tirofiban within 75 minutes after diagnosis and primary PCI was 60% lower than those receiving dual anti-platelet therapy.

Is this time-delay concept the same for other anti-platelet agents such as clopidogrel? ‘The answer is yes.^4^ If clopidogrel is given within six hours there is a significant benefit on death, re-MI or stroke in patients undergoing primary PCI, which disappears if clopidogrel is given more than 12 hours after diagnosis’, Prof Valgimigli said.

Is there benefit from using GIIb/IIIa in non-STEMI patients? ‘In a recent metaanalysis of 14 RCTs, including 3 424 patients,^5^ of tirofiban on top of clopidogrel, and aspirin versus clopidogrel plus aspirin, tirofiban reduced death/MI in the 30 days post-intervention, with an NNT of 16 to save one life or prevent one MI.’ If one looks at the background therapy and asks if clopidogrel is improving the effect of tirofiban, the answer is apparently negative.^5^

‘Overall, the explanation for the added value of GIIb/IIIa might lie within the multiple other pathways of platelet activation which still function in the presence of aspirin and clopidogrel – the platelet aggregation inhibitor action of the GIIb/ IIIas is therefore a necessary and useful action’, Prof Valgimigli concluded.
